# Supplementary material for: Reconciling Mining with the Conservation of Cave Biodiversity: A Quantitative Baseline to Help Establish Conservation Priorities
Source: PLoS One. 2016 Dec 20;11(12):e0168348. doi: 10.1371/journal.pone.0168348 (PMC5173368; doi:10.1371/journal.pone.0168348)
Supplement: S1 Dataset — (ZIP) [file pone.0168348.s002.zip › Taxa/Serra Norte/SN_2013/N5S/Lista N5S-58.pdf]

CAVIDADE N5S-0058

Classe	Ordem	Fam/Outros	Gên/Outros	Espécie	S-U	U-S	Zon.
Arachnida	Amblypygi	Phrynidae	*Heterophrynus*	*longicornis*	4	2	E
Arachnida	Araneae	Araneidae	*Alpaida*	aff. *negro*		X	E
Arachnida	Araneae	Corinnidae		jovem		2	E
Arachnida	Araneae	Ochyroceratidae		jovem		X	E
Arachnida	Araneae	Pholcidae	*Mesabolivar*	*aurantiacus*	X	X	E
Arachnida	Araneae	Pholcidae	*Mesabolivar*	sp.1	X	X	E
Arachnida	Araneae	Theridiosomatidae	*Plato*	sp.1		X	E
Arachnida	Opiliones	Cosmetidae	*Roquettea*	*singularis*		1	E
Arachnida	Opiliones	Sclerosomatidae	*Prionostema*	sp.1		5	E
Chilopoda	Geophilomorpha	Geophilidae		sp.1	1		E
Entognatha	Collembola	Paronellidae		sp.1		X	E
Entognatha	Collembola	Paronellidae		sp.6	X		E
Insecta	Blattodea	Blattidae		sp.5	1		E
Insecta	Blattodea	Polyphagidae		sp.1		1	E
Insecta	Coleoptera	Ptiliidae		jovem	X	X	E
Insecta	Diptera	Cecidomyiidae	Cecidomyiinae	sp.	X	X	E
Insecta	Diptera	Chaoboridae		sp.	X		E
Insecta	Diptera	Chironomidae		sp.	X		E
Insecta	Diptera	Dolichopodidae		sp.	X		E
Insecta	Diptera	Psychodidae	Phlebotominae	sp.	X	X	E
Insecta	Diptera	Tipulidae	Tipulinae	sp.	X	X	E
Insecta	Homoptera	Cixiidae		jovem		X	E
Insecta	Hymenoptera	Formicidae	*Gnamptogenys*	sp.1	X	X	E
Insecta	Hymenoptera	Formicidae	*Pachycondyla*	sp.2		X	E
Insecta	Hymenoptera	Formicidae	*Pheidole*	sp.3	X	X	E
Insecta	Hymenoptera	Formicidae	*Pheidole*	sp.4	X		E
Insecta	Hymenoptera	Vespidae		sp.1	X		E
Insecta	Lepidoptera	Gelechioidea		sp.		X	E
Insecta	Lepidoptera	Noctuoidea		sp.5		2	E
Insecta	Lepidoptera	Tineoidea		sp.1		X	E
Insecta	Orthoptera	Phalangopsidae	*Paraclodes*	sp.	8	2	E
Insecta	Orthoptera	Phalangopsidae	*Phalangopsis*	sp.		4	E
Insecta	Thysanura	Nicoletiidae		sp.1	X	X	E
Malacostraca	Isopoda	Philosciidae		sp.1		X	E
Amphibia	Anura			sp.	2	1	E
Mammalia	Rodentia	Cricetidae	*Rhipidomys*	sp.		1	E
